# Case Report: Successful conversion of gallbladder cancer with intrahepatic metastases using camrelizumab combined with gemcitabine and cisplatin

**DOI:** 10.3389/fimmu.2026.1724263

**Published:** 2026-05-19

**Authors:** Dongdong Wang, Yongzhao Li, Gengyuan Shi, Wei Wang, Tianlong Ma, Chen Mi, Yongyue Du, Siyang Wang, Xiyang Sheng, Longbo Wang, Xuechao Xu, Yun Wang, Hanteng Yang

**Affiliations:** 1 Department of General Surgery, Lanzhou University Second Hospital, Lanzhou, Gansu, China; 2 Department of Oncology, Gansu Provincial Second People’s Hospital, Lanzhou, Gansu, China

**Keywords:** camrelizumab, conversion therapy, gallbladder cancer, immunotherapy, surgery

## Abstract

**Background:**

Gallbladder cancer is an extremely aggressive malignant tumor of the digestive system. It presents with no obvious symptoms in its early stages, meaning most patients are already in advanced stages at diagnosis. The response to neoadjuvant therapy for gallbladder cancer is poor, and the majority of patients with advanced gallbladder cancer cannot achieve tumor downstaging through drug treatment to become eligible for surgery. This case focuses on discussing the selection of immunotherapy drugs and the determination of their treatment cycles.

**Case summary:**

This case report describes a patient with gallbladder cancer and intrahepatic metastases. Following contrast-enhanced computed tomography (CECT) evaluation, R0 resection of the lesions was deemed unfeasible. The patient underwent conversion therapy with camrelizumab combined with gemcitabine and cisplatin. The patient responded well to this regimen, with rapid tumor shrinkage after three cycles of treatment. Grade IV neutropenia developed during therapy but resolved without serious consequences following prompt intervention. After reassessment, the patient underwent open cholecystectomy combined with segmental resection of the liver segments IVb/V, lymph node dissection, and high hepaticojejunal anastomosis. Postoperative recovery was favorable, with PFS reaching 18 months.

**Conclusion:**

The regimen of camrelizumab combined with gemcitabine and cisplatin demonstrated potential conversion therapy value in this patient with advanced gallbladder cancer. However, its definitive efficacy and the specific role of camrelizumab still require validation through large-scale clinical trials.

## Introduction

Gallbladder cancer is a highly invasive malignant tumor of the biliary tract with a short median survival period (1, 2), and an average survival of only 6 months (3). Although its incidence is low, it is common in some countries in South America, India, and Western Europe (4), predominantly affecting women and the elderly. Gallstones represent the primary risk factor (5, 6). Gallbladder cancer lacks specific early clinical manifestations, with most patients diagnosed at an advanced stage. Resection of the lesion + local resection of potentially residual disease + lymph node dissection represents the optimal treatment approach. For patients with high-risk factors for early recurrence, neoadjuvant therapy may be administered preoperatively to achieve R0 resection (7). However, even among surgically treated patients, the 5-year overall survival rate remains unfavorable, ranging from 5% to 26% (8). Consequently, the overall treatment outcomes for gallbladder cancer remain suboptimal.

Through reviewing relevant literature, we learned that gallbladder cancer exhibits poor sensitivity to radiotherapy and chemotherapy, and the efficacy of immunotherapy and targeted therapy remains limited (9). Cases where conversion therapy successfully converted advanced gallbladder cancer to meet surgical criteria are rare. We now report a case where chemotherapy combined with immunotherapy successfully converted advanced gallbladder cancer to meet surgical criteria. The patient demonstrated high treatment sensitivity, did not receive adjuvant therapy postoperatively, and achieved favorable outcomes: no signs of tumor recurrence at 11 months post-surgery, with Progression-Free Survival (PFS) reaching 18 months.

## Case presentation

**Figure 1:**
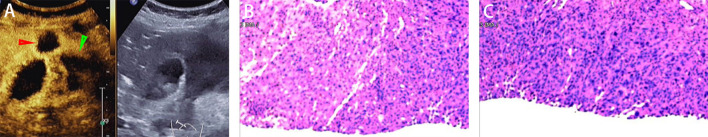
Contrast-enhanced ultrasound of the liver and liver biopsy results. **(A)** Contrast-enhanced ultrasound revealed a mixed-echo lesion approximately 5.4×4.3 cm in size adjacent to the gallbladder, with an ill-defined border from the gallbladder wall. Scattered punctate blood flow signals were observed around the lesion. Additionally, a hypoechoic lesion approximately 2.2×2.1 cm in size was detected in liver segment S5, showing heterogeneous rim enhancement with clear borders and regular morphology. **(B, C)** Pathological sections from the liver biopsy indicated moderately to poorly differentiated adenocarcinoma. Based on the patient’s history, this is consistent with metastatic gallbladder adenocarcinoma to the liver.

**Figure 2:**
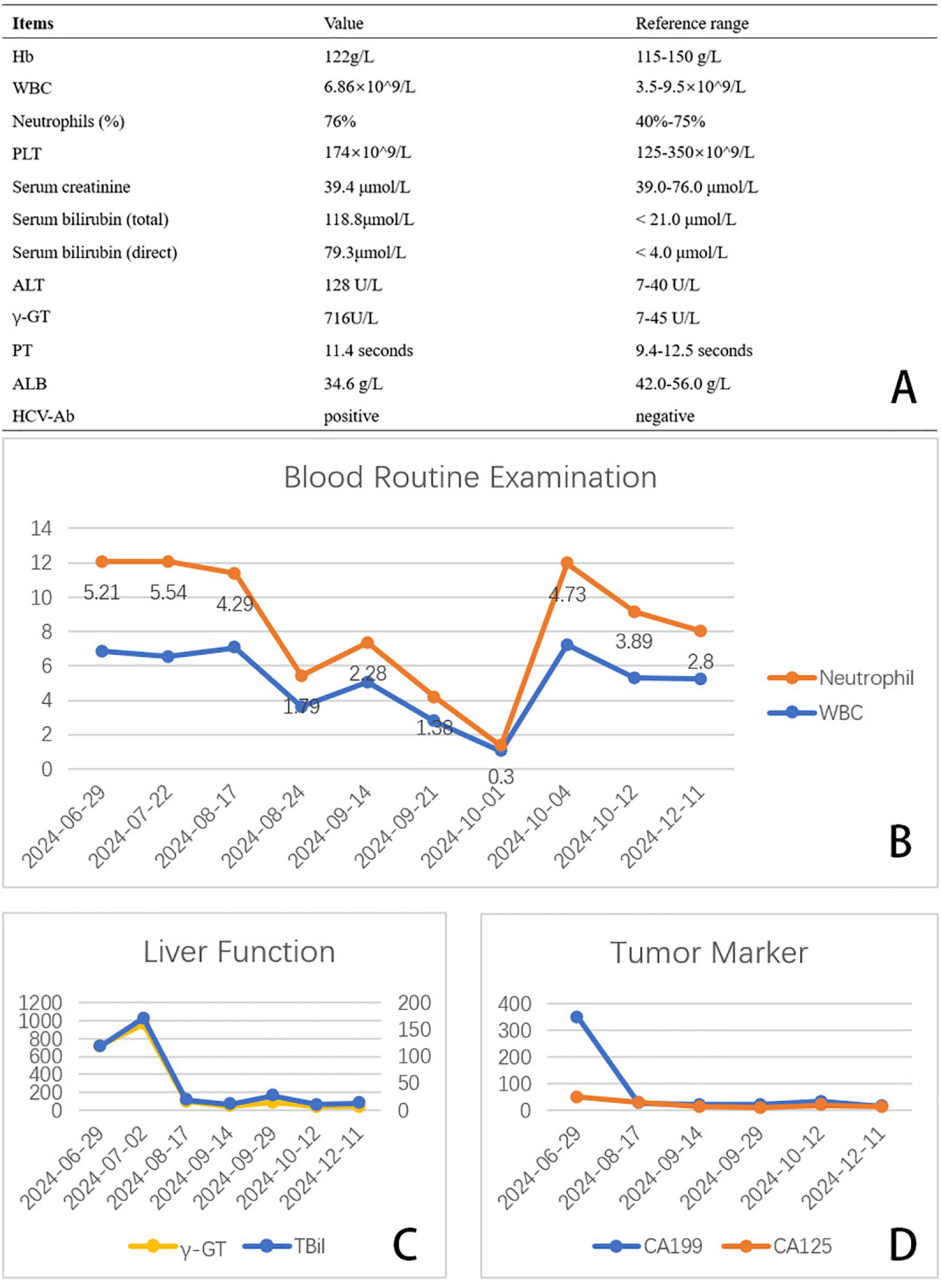
**(A)** Admission laboratory test results. **(B-D)** Trends in laboratory test results over time during treatment.

**Figure 3:**
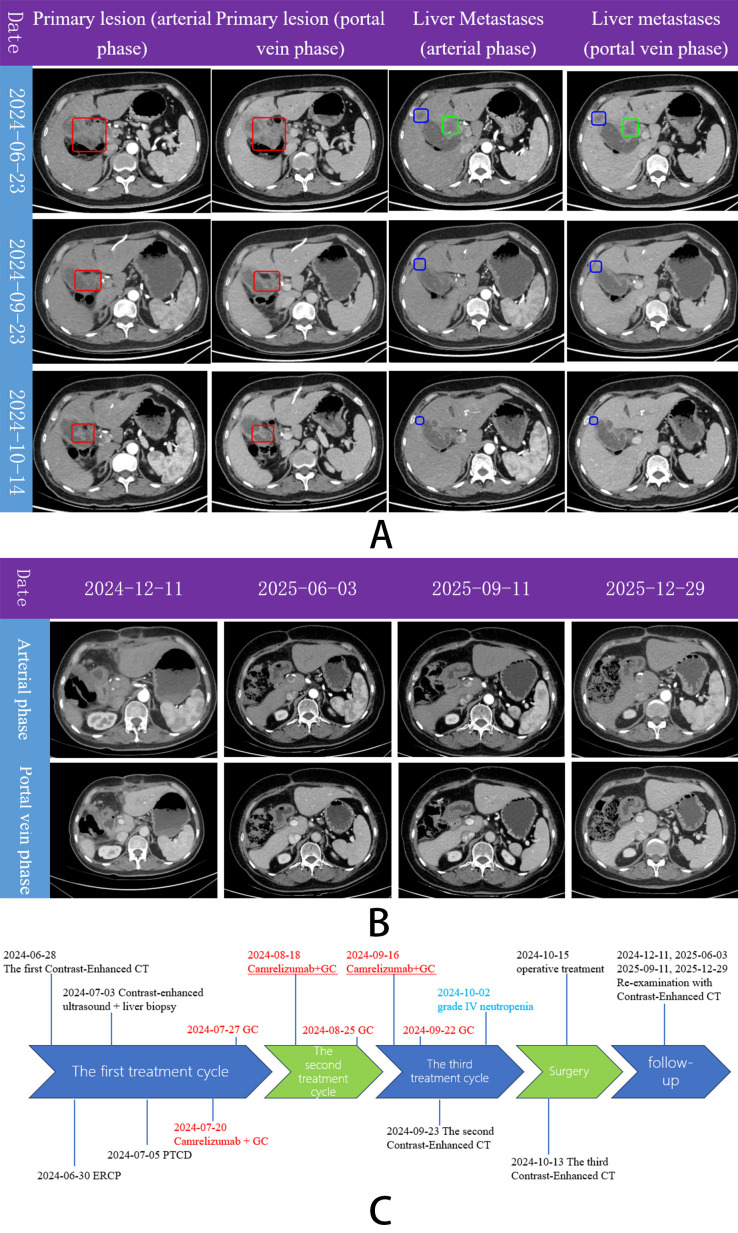
Abdominal enhanced CT scan and clinical timeline. **(A)** On three preoperative contrast-enhanced CT scans of the entire abdomen, the size of the primary tumor (red box) was significantly reduced in both the arterial and portal phase images, decreasing from 5.4 × 4.3 cm to 4.2 × 2.6 cm, and then to 3.3 × 2.0 cm. The size of the liver metastases (blue box) was significantly reduced in both the arterial and portal phases, decreasing from 2.0 × 1.6 cm to 1.3 × 1.0 cm, and then to 0.6 × 0.7 cm. An enlarged lymph node (green box) was visible in the hepatic hilum on the first CT scan. **(B)** Four postoperative follow-up enhanced abdominal CT scans demonstrated findings consistent with cholecystectomy for gallbladder carcinoma, with no abnormal enhancement and no evidence of lymph node enlargement. **(C)** Clinical timeline.

**Figure 4:**
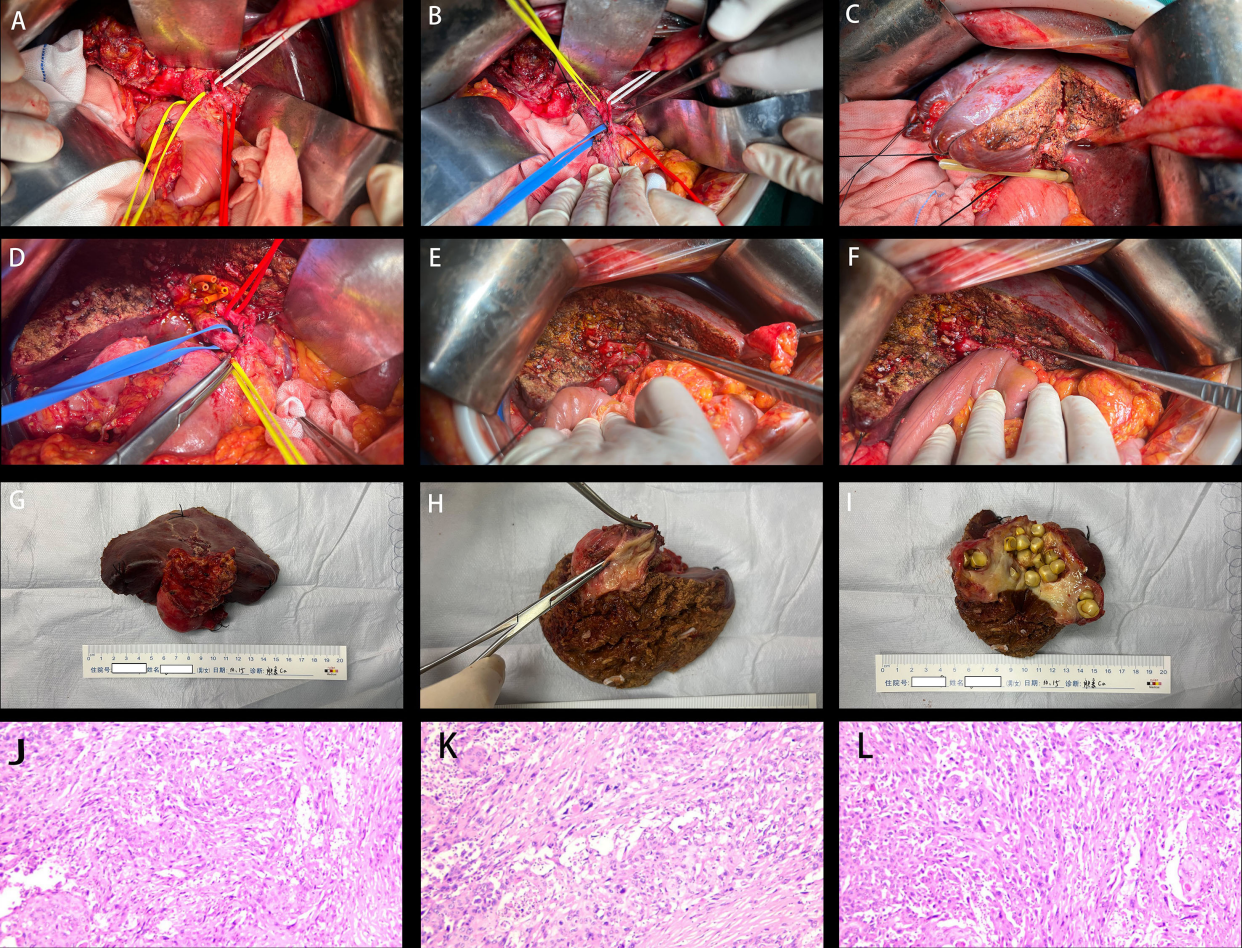
Intraoperative photographs, postoperative gross specimens, and pathological examination results. **(A)** Dissection of the common bile duct, proper hepatic artery, and cystic artery; **(B)** Dissection of the portal vein; **(C)** Hepatic segment IVb/V resection; **(D)** Placement of biliary stents to expose the intrahepatic bile ducts; **(E)** Exposure of the intrahepatic bile ducts; **(F)** Completion of hepaticojejunal anastomosis; **(G)** Overall view of the tumor; **(H)** Malignant transformation of the gallbladder mucosa; **(I)** Gallbladder calculi. **(J-L)** Histopathological examination of surgical specimen reveals (Staining: Hematoxylin and eosin; Magnification: ×100): 1. (Gallbladder) Elevated-type poorly differentiated adenocarcinoma with extensive necrosis. Cancer invades gallbladder serosa. Residual cancer tissue <50%. Tumor dimensions: 2×2×1.5 cm. Involvement of vascular, lymphatic, and neural structures. No invasion of bile ducts, left hepatic duct margins, or liver parenchyma. No cancer detected at bile duct margins. 2. (Gastric wall nodule) Fibrous connective tissue and adipose tissue with minimal inflammatory cell infiltration. Immunohistochemical (CKp) staining confirmed no evidence of carcinoma. 3. Immunohistochemical (CKp) staining confirmed no cancer metastasis in lymph nodes (0/12). Immunohistochemical staining: Cancer cells showed CK8/18 (partially positive), CK7 (positive), CK20 (negative), Her-2 (negative), p53 (mutant type), CDX-2 (negative), Syn (negative), Ki67-positive cells 70%.

A 60-year-old female presented to an external hospital with back distension and pain. An enhanced upper abdominal computed tomography (CT) scan revealed: A mass at the base of the gallbladder is most often indicative of a malignant tumor, with invasion of the adjacent peritoneum; a nodule in segment S3 of the liver; and a mass in segment S4 of the liver invading the hilar bile duct. Seeking further evaluation, the patient was admitted to our hospital. Repeat contrast-enhanced chest and abdominal CT revealed: gallbladder cancer highly suspected, multiple gallbladder stones, metastatic tumor in segment S5 of the liver, multiple metastatic lymph nodes in the hepatic hilum and hepatoduodenal ligament, invasion and stricture of the hepatic hilum bile duct, and dilatation of the intrahepatic bile ducts; no abnormalities noted in the lungs. Liver contrast-enhanced ultrasound findings: A mixed-echo lesion near the gallbladder, measuring approximately 5.4 × 4.3 cm, with irregular enhancement around the lesion and continuity with the gallbladder wall. A hypoechoic lesion in segment S5 of the liver, measuring approximately 2.2 × 2.1 cm, with irregular enhancement around the lesion, clear borders, and a regular shape (**Figure 1A**). Liver biopsy revealed moderately to poorly differentiated adenocarcinoma (**Figures 1B, C**). Combined with the history, liver metastasis from gallbladder adenocarcinoma was suspected. Following a multidisciplinary team (MDT) discussion, we concluded that there is a clear demarcation between the lesion in segment S5 of the liver and the primary gallbladder tumor on imaging, with no continuity between the two. This is therefore considered a metastatic lesion. The diagnosis is gallbladder cancer with liver metastasis, cT3N1M1, stage IVB. (AJCC Gallbladder Carcinoma Staging, 8th Edition). Admission laboratory results indicated abnormal liver function and obstructive jaundice (**Figure 2A**). Immediate endoscopic retrograde cholangiopancreatography (ERCP) with biliary stent placement was performed for jaundice relief. Following stent placement, the patient’s total bilirubin continued to rise to 171.6 μmol/L. We considered that the biliary stent was not positioned at the obstruction site, potentially worsening biliary obstruction. Subsequently, percutaneous transhepatic cholangiodrainage (PTCD) was performed for jaundice relief, resulting in significant improvement of the patient’s jaundice. The patient presented with advanced gallbladder cancer inoperable for R0 resection. The patient received conversion therapy according to the following regimen: the patient received 200 mg camrelizumab intravenously every 3 weeks (on day 1 of the cycle) for up to eight cycles in combination with 1000mg/m^2^ gemcitabine and 25 mg/m^2^ cisplatin intravenously on days 1 and 8 every 3 weeks for up to eight cycles. Prior to the start of chemotherapy, 20 mg of diphenhydramine hydrochloride was administered intramuscularly and 20 mg of cimetidine was administered intravenously. Metoclopramide hydrochloride 10 mg was administered intramuscularly both before and after chemotherapy. Following chemotherapy, 1000 mL of balanced salt solution was administered for rehydration to mitigate drug-induced nephrotoxicity. The doses of camrelizumab, gemcitabine, and cisplatin remained unchanged throughout the treatment. Following the first treatment cycle, the patient’s tumor marker levels decreased significantly, with CA199 dropping from 350 U/mL to 28 U/mL (**Figure 2D**). After three treatment cycles, the patient’s tumor burden was substantially reduced (**Figure 3A**). During the third treatment cycle, the patient developed neutropenia, which improved after administration of human granulocyte colony-stimulating factor (G-CSF). Following discharge, the patient developed severe bone marrow suppression with a neutrophil count dropping to 0.3×10^9/L (**Figure 2B**), classified as Grade IV neutropenia (per WHO criteria for acute and subacute toxicities of antineoplastic agents). Upon admission to our hospital, immediate symptomatic treatment with human granulocyte colony-stimulating factor injection was initiated, and the patient was discharged after recovery. After three cycles of treatment, the patient underwent open cholecystectomy combined with resection of liver segments IVb/V, lymphadenectomy, and high hepaticojejunostomy at our hospital. Intraoperative photographs are shown below (**Figure 4**). The procedure proceeded smoothly. The gross specimen is shown in the figure (**Figure 4**). The patient recovered and was discharged one month postoperatively. Pathological examination revealed: Poorly differentiated adenocarcinoma of the gallbladder with necrosis (**Figure 4**). No adjuvant therapy was administered postoperatively. Follow-up enhanced abdominal CT scans at 2, 7, 11 and 14 months postoperatively showed no evidence of recurrence (**Figure 3B**).

## Discussion

Gallbladder cancer is a relatively rare malignant tumor of the digestive system, characterized by high invasiveness and poor prognosis. Key risk factors for gallbladder cancer include central obesity (29.7%), gallbladder stones (27.9%), and lack of physical activity (20.5%). Gallbladder cancer lacks specific symptoms and lacks specific diagnostic markers. The most common symptoms of early gallbladder cancer are indigestion and abdominal pain. Laboratory test results generally show abnormal liver function or abnormal tumor markers, such as elevated CA-199 and CEA levels (10), but these are common in gastrointestinal tumors. For early-stage gallbladder cancer, surgical resection remains the only curative treatment option. The direct drainage of the gallbladder vein into the portal venous system facilitates tumor invasion into adjacent organs (11), leading to early metastasis. Consequently, most patients (>70%-90%) present at a stage where surgical intervention is no longer feasible. For these patients, conversion therapy represents the sole therapeutic hope.

The combination of gemcitabine and cisplatin (GC) is the standard first-line regimen in conversion therapy, but the response rate to this combination chemotherapy is only approximately 30%. The ABC-02 trial demonstrated that the gemcitabine plus cisplatin regimen yields suboptimal outcomes for locally advanced or metastatic gallbladder cancer, with a median overall survival of only 11.7 months and a median PFS of 8 months (12). However, it outperformed the gemcitabine monotherapy group. The recent emergence of immunotherapy offers new hope for patients with advanced gallbladder cancer. In September 2022, the U.S. FDA approved the TOPAZ-1 clinical trial, comparing the efficacy of durvalumab plus GC versus placebo plus GC. Results demonstrated significantly prolonged overall survival in the durvalumab group (median overall survival: 12.9 months [95% CI 11.6–14.1] vs. 11.3 months [10.1–12.5]; hazard ratio 0.76 [95% CI 0.64–0.91]). The most common Grade 3 or 4 treatment-related adverse events during the trial were low neutrophil count (70 [21%] vs 86 [25%]), anemia (64 [19%] vs 64 [19%]), and neutropenia (63 [19%] vs 68 [20%]) (13). The KEYNOTE-966 clinical trial, which began in September 2019, compared the efficacy of pembrolizumab combined with GC against placebo combined with GC. The results showed that the pembrolizumab group had a better overall survival benefit (median OS of 12.7 months [95% CI 11.5–13.6 months] vs. 10.9 months [9.9–11.6]; hazard ratio 0.83 [95% CI 0.72–0.95]) (14). Immunotherapy has demonstrated significant survival benefits for unresectable gallbladder cancer. PD-L1 expression has been confirmed to be useful for screening patients who may respond to ICIs. The roles of TMB and MSI as biomarkers in predicting the effectiveness of immunotherapy for certain cancers have also been established (15). It is recommended to perform PD-L1 testing before formulating an immunotherapy regimen for gallbladder cancer patients. If necessary, TMB and MSI testing may also be conducted to predict whether the patient will have a favorable response to immunotherapy (16).

In the ABC-02 trial, the complete response rate in the GC group was only 0.6%, and the partial response rate was 25.5% (12). This suggests that the likelihood of achieving R0 resection with the GC regimen is low, and patients may not be eligible for surgical treatment. Although the TOPAZ-1 and KEYNOTE-966 trials have established the combination of immune checkpoint inhibitors (ICIs) and GC chemotherapy as a first-line treatment for advanced biliary tract cancer (BTC), achieving R0 resection through conversion therapy remains a significant clinical challenge. In the TOPAZ-1 trial, the objective response rate (ORR) was 26.7%, and the median PFS was 7.2 months (17). The KEYNOTE-966 trial reported an ORR of 29% and a median PFS of 6.5 months (14). In these cohorts of advanced patients, conversion surgery was rarely mentioned. The patient in this case responded very rapidly to the combination therapy. After the first cycle, CA199 decreased from 350 U/mL to 28 U/mL, and the patient met the criteria for R0 resection after only three cycles of combination therapy. Furthermore, the patient’s PFS has now reached 18 months, which is 2–3 times longer than the median PFS observed in the aforementioned clinical trials. This suggests that certain subtypes of gallbladder cancer may exhibit exceptional sensitivity to immunotherapy.

This rapid response may be associated with the induction of immunogenic cell death (ICD) in tumor cells by chemotherapeutic agents. Cisplatin induces the release and exposure of damage-associated molecular patterns (DAMPs), leading to ICD in tumor cells, and can modulate the infiltration of immune cells into the immune microenvironment. This enhances antitumor immunity and exerts a synergistic effect with immune checkpoint inhibitors (ICIs) (18). Gemcitabine enhances the antigen-presenting capacity of tumor cell MHC class I molecules, improving the recognition of tumor antigens, and can selectively deplete myeloid-derived suppressor cells (MDSCs), thereby enhancing antitumor immunity (18). This differential response may also be related to the unique pharmacological properties of the ICIs used. Durvalumab binds to PD-L1, whereas pembrolizumab and camrelizumab are both PD-1 inhibitors. However, camrelizumab binds to the C, C’, and FG loops of the PD-1 molecule, whereas pembrolizumab binds to the CD loop, and their pharmacodynamic properties also differ (19). Given its high accessibility, favorable cost-effectiveness, and the growing body of evidence in Chinese biliary tract cancer (BTC) cohorts, camrelizumab represents a highly promising alternative. The rapid reduction in tumor burden and subsequent long-term survival observed in this patient warrant further investigation into whether camrelizumab’s specific binding characteristics contribute to enhancing its synergistic effect with conventional chemotherapy for gallbladder cancer.

After admission, the patient in this case developed obstructive jaundice. We initially performed PTCD for drainage and administered hepatoprotective therapy. Once the jaundice and liver function improved, a MDT assessment determined that R0 resection of the tumor was not feasible. Given that the patient’s Performance Status (PS) score was ≤1 and they were deemed able to tolerate chemotherapy, we decided to initiate conversion therapy with a combination of immune checkpoint inhibitors (ICIs) and a chemotherapy regimen, with the goal of eventually enabling the patient to undergo surgical treatment. When it comes to selecting ICIs, pembrolizumab (KEYNOTE-966) and durvalumab (TOPAZ-1) have demonstrated excellent efficacy in the treatment of advanced gallbladder cancer. However, both drugs are prohibitively expensive, imposing a significant financial burden on many patients. Camrelizumab, a humanized PD-1 monoclonal antibody independently developed and owned by Jiangsu Hengrui Medicine, has been approved for eight indications across five major tumor types—lung cancer, liver cancer, esophageal cancer, nasopharyngeal carcinoma, and lymphoma—since its market launch in May 2019. Compared to pembrolizumab and durvalumab, the annual treatment cost for patients is significantly reduced. Furthermore, clinical studies have demonstrated that camrelizumab exhibits favorable antitumor effects in advanced gallbladder cancer (20–23). Given the patient’s family’s limited financial resources, and after thorough discussion with the patient, the patient agreed to our recommendation of the camrelizumab plus GC regimen.

In this case, the patient demonstrated favorable responsiveness to the combination therapy. After three cycles, the tumor showed marked regression, rapidly transforming from an unresectable to a potentially R0-resectable state. This achieved the goal of conversion therapy in reducing tumor stage, creating favorable conditions for surgical intervention. However, whether the therapeutic effect in this regimen was attributable to camrelizumab requires further clinical trials to confirm. The primary adverse reaction observed during treatment was neutropenia. Following the third treatment cycle, the patient developed severe bone marrow suppression with Grade IV neutropenia. Administration of human granulocyte colony-stimulating factor (G-CSF) resolved the adverse reaction. This underscores the necessity for continuous monitoring of laboratory parameters—including complete blood counts and hepatic/renal function—during this regimen to prevent adverse outcomes due to delayed detection of related toxicities. This patient underwent surgery after three cycles of treatment due to severe bone marrow suppression. This decision was made after weighing the current therapeutic efficacy against the risks associated with continuing treatment. However, the optimal number of treatment cycles remains undetermined, as it requires balancing tumor regression with drug toxicity.

Although this case demonstrated remarkably significant therapeutic outcomes, this report has certain limitations. First, this study is a single-case report, representing a very low level of evidence. Given patient-to-patient variability and tumor heterogeneity, the generalizability and reproducibility of this treatment regimen remain to be determined. Second, comprehensive biomarker testing of the patient’s tumor tissue—including PD-L1 expression levels, TMB, and MSI status—was not performed before and after treatment. This lack of data limits our ability to investigate, at the molecular and genetic levels, the mechanisms underlying the patient’s rapid immune response to the combination of camrelizumab and the GC regimen.

## Conclusion

The diagnosis and treatment of gallbladder cancer face numerous challenges. Surgery remains the optimal therapeutic intervention for gallbladder cancer, as it can significantly prolong patient survival. This case demonstrates the potential value of camrelizumab in conversion therapy for patients with advanced gallbladder cancer. However, as a single case report, the level of evidence is very low, and key biomarker data are lacking to elucidate the underlying response mechanism. Therefore, whether camrelizumab truly exerts a therapeutic effect requires further validation through clinical trials.

## Data Availability

The original contributions presented in the study are included in the article/supplementary material. Further inquiries can be directed to the corresponding authors.
